# The role of dorsolateral and ventromedial prefrontal cortex in the processing of emotional dimensions

**DOI:** 10.1038/s41598-021-81454-7

**Published:** 2021-01-21

**Authors:** Vahid Nejati, Reyhaneh Majdi, Mohammad Ali Salehinejad, Michael A. Nitsche

**Affiliations:** 1 Department of Psychology, Shahid Beheshti University, Tehran, Iran; 2 Department of Psychology, Refah University, Tehran, Iran; 3 Department of Psychology and Neurosciences, Leibniz Research Centre for Working Environment and Human Factors, Dortmund, Germany; 4 International Graduate School of Neuroscience, Ruhr-University Bochum, Bochum, Germany; 5 Department of Neurology, University Medical Hospital Bergmannsheil, Bochum, Germany

**Keywords:** Perception, Prefrontal cortex

## Abstract

The ventromedial and dorsolateral prefrontal cortex are two major prefrontal regions that usually interact in serving different cognitive functions. On the other hand, these regions are also involved in cognitive processing of emotions but their contribution to emotional processing is not well-studied. In the present study, we investigated the role of these regions in three dimensions (valence, arousal and dominance) of emotional processing of stimuli via ratings of visual stimuli performed by the study participants on these dimensions. Twenty- two healthy adult participants (mean age 25.21 ± 3.84 years) were recruited and received anodal and sham transcranial direct current stimulation (tDCS) (1.5 mA, 15 min) over the dorsolateral prefrontal cortex (dlPFC) and and ventromedial prefrontal cortex (vmPFC) in three separate sessions with an at least 72-h interval. During stimulation, participants underwent an emotional task in each stimulation condition. The task included 100 visual stimuli and participants were asked to rate them with respect to valence, arousal, and dominance. Results show a significant effect of stimulation condition on different aspects of emotional processing. Specifically, anodal tDCS over the dlPFC significantly reduced valence attribution for positive pictures. In contrast, anodal tDCS over the vmPFC significantly reduced arousal ratings. Dominance ratings were not affected by the intervention. Our results suggest that the dlPFC is involved in control and regulation of valence of emotional experiences, while the vmPFC might be involved in the extinction of arousal caused by emotional stimuli. Our findings implicate dimension-specific processing of emotions by different prefrontal areas which has implications for disorders characterized by emotional disturbances such as anxiety or mood disorders.

## Introduction

The prefrontal cortex (PFC) consists of about two-thirds of the human frontal cortex and is involved in various aspects of behavioral management. Anatomically, the prefrontal cortex is divided into three main areas, including the dorsolateral prefrontal cortex (dlPFC), the medial prefrontal cortex (mPFC), and the ventral, inferior or orbital frontal cortex (OFC). Functionally and structurally, the two latter areas are highly interconnected and often considered as a more or less uniform structure, the ventromedial prefrontal cortex (vmPFC)^1^.

Functionally, the dlPFC is mainly involved in executive functioning and cognitive control^2–4^. Here, it contributes to a large variety of psychological processes, such as working memory^5^, divergent thinking^6^, executive attention^4, 7^, and decision making^8^. On the other hand, the vmPFC is sensitive to the reward or value of stimuli^9, 10^, value-based decision-making^11^, anticipation of reward^12^ and self-based evaluation^13^. To put it in a nutshell, the vmPFC is assumed to have a crucial role in emotional processing, whereas the dlPFC is predominantly involved in cognitive control and executive processing.

It is however debatable if such a strict functional distinction of the respective areas does hold. On the one hand, concepts about the relation between cognition and emotion have a long, and confusing history in philosophy and psychology. Respective concepts have ranged from more or less complete independence of cognition and emotion^14^ to a interaction with primacy of affect^15^, or cognition^16^. In general agreement with the interaction hypothesis, functional imaging studies show a gradual transition between ventromedial, and dorsolateral prefrontal areas with regard to emotion processing^17^.

Beyond the simultaneous contribution of both, dlPFC and vmPFC areas in emotional processing, specific concepts are available proposing that and how these areas interact to handle emotional experiences. Two main mechanisms for interaction between emotion and cognition are considered as modes of communication between vmPFC and dlPFC. First, emotional stimuli serve as modulators of executive functions. Emotionally high-valenced information can capture attentional resources and thus direct or change the outcomes of executive functions. In other words, attributing emotional value to some stimuli will improve their chance being selected for further processing^18^. Second, there is a regulating or controlling role of cognitive processing on emotional processing, labeled reappraisal or emotion regulation^19, 20^. Onset, duration, intensity, or content of emotional response are regulated by executive functions. Hence, any deficit in executive functions results in impairment of emotion regulation, as found in attention deficit- hyperactivity disorder (for a review see^21^).

The interaction between these PFC compartments has an important impact on behavior. One well-documented role of the dlPFC is maintenance and regulation of top-down control for driving appropriate behavior^22, 23^. Respective prefrontal control deficits are tightly connected to psychopathology as well as treatment procedure in disorders with executive dysfunctions^24, 25^. Hyperactivity of the vmPFC, and the amygdala due to insufficient control via the dlPFC leads to an attention bias to threat-related stimuli in anxiety^26^. In the same way, a lack of regulatory control of the dlPFC on dysregulated fear circuits in the vmPFC is involved in post-traumatic stress disorder (PTSD)^27^ which comes with cognitive deficits in both emotional and non-emotional materials^28^. Reduced dlPFC control over the vmPFC or an imbalance of respective interactions is furthermore associated with depression^29, 30^.

In most available studies, the valence of emotional stimuli has been studied as a decisive factor in the interaction between cognition and emotion, and the respective contribution of the dlPFC and vmPFC. vmPFC and dlPFC are interacting in hot and cold executive functions^31^. Furthermore, positive affect improves executive functioning^32^, and psychological well-being is associated with vmPFC activity in response to negative stimuli^33^, but dlPFC activity in response to positive stimuli^34^. These results argue for the involvement of both areas in valenced-based information processing, which might be specific for emotional quality. In further accordance, the dlPFC is involved in the evaluation of the valence of pleasant pictures^35^. The role of the vmPFC in the processing of valence-specific processing of emotional content remains however more unclear. In contrast, it has a distinctive function in arousal attribution^36^. In light of these findings, the specific role of vmPFC and dlPFC in emotional processing remains a matter of debate.

Non-invasive brain stimulation methods provide an opportunity for the investigation of the causal relationship between cortical structure and various cognitive / emotional functions in both healthy and clinical populations^37–41^. In the present study, we used transcranial direct current stimulation (tDCS) to explore the role of dlPFC and vmPFC in the perception of emotional dimensions. This technique modulates cerebral neuronal activity, and excitability by applying a weak direct electrical current to the brain through the scalp. Depending on the current flow direction, the electrical current between the anode and cathode electrodes results in an increase or decrease of cortical excitability underneath the respective electrodes. Within certain limits, tDCS with the anode positioned over the target area enhances cortical excitability, and cathodal tDCS reduces it at the macroscopic level^42^ although there are unexpected effects outside standard protocols of tDCS^43^. The primary effect during stimulation is thought to be caused by respective subthreshold membrane polarization effects, while after-effects, which can last for an hour or more after the intervention, depend on strengthening or weakening of glutamatergic synapses in the presence of downregulation of GABA^44, 45^.

Earlier tDCS studies have investigated the role of the dlPFC in emotional processing separately in the light of laterality. Based on these studies in healthy individuals, anodal tDCS over the left dlPFC resulted in enhanced positive mode^46–48^ and decreased negative mood^49, 50^ while over the right dlPFC led to mood dysregulation and negative emotional processing^51^. Imbalance in this lateralized emotional processing has been considered as a hallmark of depression^52^ and therefore using anodal right and cathodal left dlPFC stimulation is used for amelioration of depressive symptoms^25^. A recent combined tDCS and pupillometry study in healthy individuals found the role of the left dlPFC in processing of negative emotional images, while the right vmPFC decreases the reaction to emotional images irrespective of valence^53^. Together, these studies suggests the left dlPFC is a more important structure in emotional processing and regulation.

However, modulatory effects of tDCS in cognitive and behavioral domains have not been as consistent as for the motor areas^54, 55^ and thus it might not be reasonable to hold a linear, simplistic assumption for effects of tDCS on cognition and behavior. Stimulation parameters including stimulation intensity, duration polarity and even task-specific features^56, 57^ are important determinates of cognitive effects of tDCS. For example, a recent study reported that while 1 mA tDCS (anodal, cathodal, sham) did not affect top-down control 2 mA cathodal tDCS significantly improved it^58^ or in another study the opposite or bidirectional effect of anodal vs cathodal tDCS was not observed at the behavioral level^59^. Taking these considerations into account, in this study, we aimed to determine the respective roles of the dlPFC and vmPFC in the attribution of valence, arousal and dominance to emotional stimuli via an increase of their excitability through anodal tDCS. We hypothesized that upregulation of the dlPFC, and vmPFC via anodal tDCS might alter emotion processing if the respective areas are involved. Specifically, we expected that both areas might contribute at least gradually differently to the respective emotional dimensions and that the dlPFC and vmPFC are relevant for the attribution of valence and arousal, respectively.

## Experimental procedures

### Participants

Twenty-two right-handed university students aged between 20–30 years (mean 25.21 ± 3.84; 4 men, 18 women), which were blinded to the study hypothesis, took part in the experiment. All participants were university students, native speakers, and had normal or corrected-to-normal vision. We used G*power^60^ to determine the required sample size. Results showed that based on a power of 0.95, an alpha level of 0.05, and a medium effect size (f = 0.40) suggested for tDCS studies^61^, the required sample size for our design (repeated-measures ANOVA with 3 measurements) is 18. We added four more subjects to compensate for drop-outs, and unforeseen variability. Participants had no history of head injury or surgery, drug abuse or dependence, psychiatric or neurological disorders, and were not pregnant. All participants signed a written informed consent form after the aim and procedure of the study were explained, and were free to withdraw from the experiment at any stage. The experimental procedure was approved by the ethics committee for research involving human participants at the neurocognitive laboratory at the Shahid Beheshti University and the study was conducted in accordance with the latest version of the Declaration of Helsinki. All participants signed a written informed consent form before participation. The authors attest to informed consent for both study participation and publication of identifying information/images for publication.

### Emotional picture rating task

**Figure 1:**
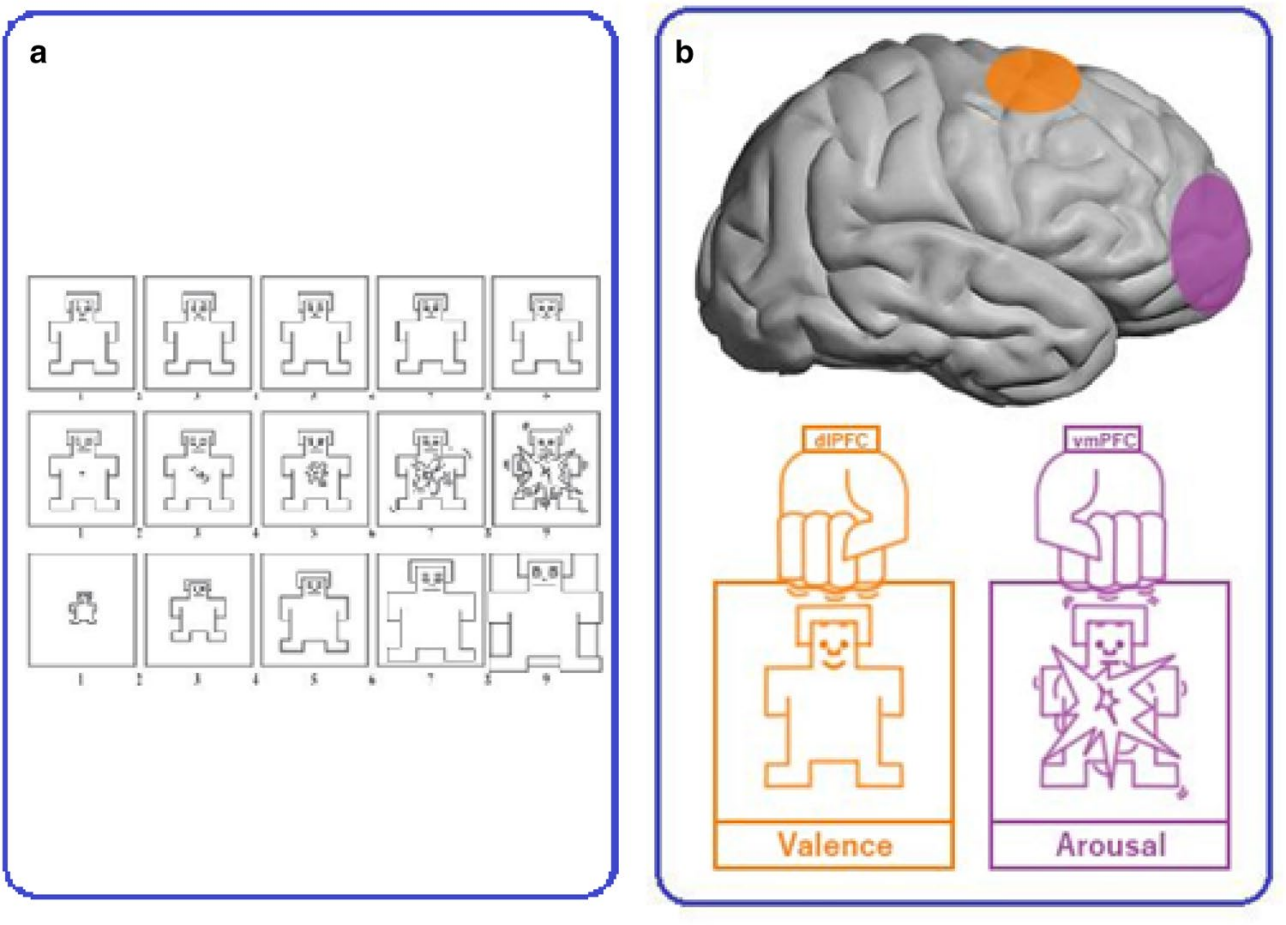
(**a**) The SAM scales for rating stimulus valence, arousal, and dominance, in order from top to bottom; (**b**) a schematic diagram for the effect of the dlPFC and vmPFC on the valence and arousal based on the findings of the present study. Abbreviations: dlPFC: dorsolateral prefrontal cortex, vmPFC: ventromedial prefrontal cortex.

In this task, images appear on the screen together with a self-assessment manikin (SAM). SAM includes 3 rating scales ranging from 1 to 9 for evaluation of valence, arousal, and dominance (Fig. 1a). SAM is a non-verbal pictorial assessment technique consisting of a graphic figure depicting a 9-point scale of valence, arousal and dominance and participants are asked to place an "X" over any of the five figures in each scale, or between any two figures, which resulted in a 9-point rating scale for each dimension^62^. Larger values on this scale represent higher values on the respective emotion dimensions. For the valence rating, higher values encode for positive, medium for neutral, and low for negative valence. The images were selected from the international affective picture system (IAPS)^63^, based on normative data of their valence, arousal and dominance. Three categories were selected based on the mean of valence, arousal, and dominance, lower than 4 (low), 4 to 6 (medium), and upper than 6 (high). The number of images at low, medium and high levels were 34, 41, and 24 in valence; 20, 55, and 25 in arousal; 13, 66, and 21 in dominance respectively. The original picture numbers of the IAPS were 1112, 1230, 1240, 1390, 1505, 1645, 1650, 1935, 2101, 2205, 2206, 2230, 2272, 2278, 2279, 2301, 2309, 2312, 2383, 2399, 2400, 2410, 2446, 2455, 2456, 2458, 2490, 2516, 2520, 2590, 2600, 2695, 2700, 2780, 2810, 2830, 3300, 5470, 5535, 5621, 5626, 5629, 5970, 6000, 6800, 6837, 6930, 7002, 7011, 7013, 7044, 7046, 7054, 7092, 7137, 7211, 7405, 7476, 7484, 7487, 7650, 8030, 8034, 8080, 8121, 8161, 8163, 8170, 8178, 8185, 8186, 8190, 8191, 8193, 8206, 8251, 8300, 8341, 8492, 8499, 9000, 9001, 9046, 9080, 9220, 9280, 9290, 9291, 9295, 9330, 9341, 9342, 9395, 9468, 9469, 9472, 9582, 9635, 9832, 9913.

The pictures remained on the screen until the response of the participants, which was self-paced, was completed, and the inter-trial interval was 1000 ms. Each image was shown three times, in an identical order, for the separate ratings of valence, arousal, and dominance. The images were presented in their original size (1024 × 768 resolution) at the center of a 13-inch computer screen with a 1280 × 1024 resolution and at an eye- distance of approximately 50–60 cm.

The pictures were divided into three randomly ordered subsets, and subset order was randomized across participants. The task takes about 10 min to complete. Participants were first screened to ensure their willingness to view negatively valenced images and then agreed to participate following the screening procedure. All participants rated valence, arousal and dominance items using SAM displayed on a computer screen.

### tDCS protocol

The tDCS device was an “ActivaDose Iontophoresis” manufactured by Activa Tek, with a 9-V battery serving as the power source. An electrical direct current of 1.5 mA generated by the stimulator was applied through a pair of saline-soaked sponge electrodes with a size of 25 cm^2^ (current density: 0.06 mA/cm^2^) for 15 min (with 15 s ramp up and 15 s ramp down). In the present study, three tDCS electrode positions were used, including (a) anodal vmPFC (FpZ)/ cathode over the right forearm, (b) anodal left dlPFC (F3)/ cathode over the right forearm, and (c) sham stimulation with one electrode over the left dlPFC and the other on the right forearm. The reference electrode over the forearm had the same size as the target electrode positioned on the head. These electrode placements were used in previous studies for targeting the respective cortical areas^31, 64, 65^. The 10–20 EEG international system was used for electrode placement. For sham stimulation, electrical current was ramped up, and then immediately down for each 15 s at the start of intervention, to generate the same sensation as the active condition^66^. The electrodes remained on the head for 15 min also in the sham stimulation condition, equivalent to active stimulation.

### Procedure

After checking the inclusion criteria, receiving information about the study, and signing the written consent form, the participants received the task instructions. Participants were blinded to the type of stimulation they received. The order of stimulation was randomized across participants. The interval between the sessions was at least 72 h to prevent carry-over effects. Five minutes after the beginning of stimulation, participants performed the picture rating tasks, which lasted for about 10 min. Following each simulation session, participants were asked to complete a short post-stimulation survey about potential side effects of the intervention. The checklist of side effects during stimulation included 5 questions addressing “itching”, “tingling”, “burning”, “pain” and “trouble concentrating” during stimulation, which were rated on a 0–5 Likert scale, where “0” represented the absence of the respective sensation, and “5” extreme intensity.

### Data analysis

This study had a single-blind, complete crossover design. Data analyses were conducted using the statistical package SPSS for Windows, version 24 (IBM, SPSS, Inc., Chicago, IL). Normality and homogeneity of variance of the data collected from each stimulation condition were confirmed using the Shapiro–Wilk and Levin tests respectively. To explore the effect of tDCS on task performance, a repeated measure 3 × 3 analysis of variance (ANOVA) was conducted with emotional dimension (3 values: valence, arousal, and dominance), tDCS condition (3 values: anodal dlPFC, anodal vmPFC and sham), and emotional valence of the pictures (3 values: high (positive), neutral, low(negative)) as the within-subject factors. The numerical values of the rating scale served as the dependent variable. Mauchly’s test was used to evaluate the sphericity of the data and in case of violation of data sphericity, degrees of freedom were corrected using the Greenhouse- Geisser method. For significant ANOVA results, for all stimulation conditions, values were pair-wise compared via Sidak post *hoc* comparisons. Reported side effects for each active tDCS protocol vs sham tDCS were analyzed via *Student’s* paired t-test (two-tailed, *p* < 0.05). A significance level of *p* < 0.05 was used for all statistical comparisons.

## Results

All participants tolerated tDCS well. No adverse effects were reported during and after stimulation except for a mild itching, tingling and burning sensation under the electrodes during approximately the first 30 s of stimulation in each tDCS condition. The occurrence of side effects is summarized in Table 1. The perceived side effects were not significantly different between stimulation conditions, except for the item “burning sensation”, which was rated significantly higher for anodal left dlPFC tDCS (*p* = 0.037), as compared with the sham condition. A similar trend was observed for this item in the anodal vmPFC tDCS condition, as compared to sham (*p* = 0.081). A data overview including the descriptive data (i.e., means and standard deviation) for the arousal, valence and dominance ratings of the stimuli under different stimulation conditions is shown in Table 2.

**Table 1:** Reported side effects during tDCS.

tDCS session	Pain	Burning sensation	Itching sensation	Trouble concentrating	Fatigue
Anodal dlPFC	0.9 (1.28)	3.1 (1.91)	1.1 (1.63)	0.33 (0.70)	0.41 (0.69)
Anodal vmPFC	0.6 (0.84)	2.4 (2.01)	0.8 (1.31)	0.30 (0.48)	0.60 (0.84)
Sham	0.6 (1.74)	1.5 (1.64)	0.9 (1.28)	0.22 (0.44)	0.10 (0.31)
*P*_dlPFC vs sham_	0.27	**0.03**	0.55	0.68	0.16
*P*_vmPFC vs sham_	0.99	0.08	0.34	0.34	0.19

Shown are the mean intensities of reported side effects, with standard deviations in brackets. tDCS = transcranial direct current stimulation; dlPFC = dorsolateral prefrontal cortex; vmPFC = ventromedial prefrontal cortex; significant results (*p* ≤ 0.05) are highlighted in bold.

**Table 2:** Descriptive statistics (mean, standard deviation) for the effects of tDCS on different dimensions of emotional processing.

	Sham M(Sd)	vmFC stimulation M (SD)	dlPFC stimulation M (SD)
**Arousal**			
High Arousal	5.72 (2.10)	4.88 (2.61)	5.94 (1.98)
Medium Arousal	3.33 (.99)	2.90 (1.39)	3.85 (1.17)
Low Arousal	3.11 (1.28)	2.48 (1.16)	3.39 (1.22)
All images	4.05 (1.29)	3.42 (0.92)	4.39 (1.42)
**Valence**			
High Valence	6.62 (1.12)	6.65 (0.84)	6.06 (1.31)
Neutral Valence	4.10 (.89)	4.05 (0.93)	3.86 (1.31)
Low Valence	3.12 (.93)	2.83 (0.86)	3.08 (1.04)
All images	4.61 (1.69)	4.51 (1.68)	4.33 (1.50)
**Dominance**			
High Dominance	4.76 (1.48)	4.59 (1.22)	4.74 (1.41)
Medium Dominance	3.88 (1.33)	4.01 (1.53)	4.26 (1.28)
Low Dominance	3.88 (1.54)	3.42 (1.38)	3.77 (1.36)
All images	4.17 (1.55)	3.51 (1.18)	4.25 (1.24)

tDCS = transcranial direct current stimulation ; M = mean; sd = standard eviation; vmPFC = ventromedial prefrontal coretx; dlPFC = dorsolateral prefrontal cortex.

The overall ANOVA shows a significant main effect of tDCS condition (*F*_1.79_ = 4.61, *p* = 0.019, partial η^2^ = 0.180) indicating a significant overall performance difference between vmPFC (3.98 ± 0.0.17), dlPFC (4.33 ± 0.0.15), and sham stimulation conditions (4.27 ± 0.0.16). Results of the Sidak post *hoc* analyses revealed only a significant difference between dlPFC and vmPFC stimulation (*p* = 0.007). The main effect of emotional valence was also significant (*F*_(1.36)_ = 84.58, *p* < 0.001, partial η2 = 0.801), showing the expected significant difference between high (M = 5.55, SD = 0.26), neutral (M = , 3.81 SD = 0.11) and low (M = 3.22, SD = 0.14) levels of emotional valence. The respective post *hoc* tests for pairwise comparisons were significant (*p* < 0.05). Results of the ANOVA are displayed in Table 3.

**Table 3:** Results of the three-way repeated-measures ANOVA for effects of tDCS conditions (anodal vmPFC/anodal dlPFC/sham), emotional dimension (arousal, valence, dominance) and emotional valence (high, low, neutral) on emotional processing.

Task	*df*	Mean square	*F*	*p*	*η*_p_^2^
tDCS condition	1.79	7.65	4.60	≤ **0.05**	0.054
Emotional dimension	1.66	17.37	2.50	0.10	0.258
Emotion valence	1.36	427.28	84.57	≤ **0.01**	0.358
tDCS × emotional dimension	3.43	6.81	4.188	≤ **0.01**	0.075
tDCS × emotional valence	2.98	1.11	1.63	0.19	0.083
Emotional dimension × emotional valence	3.31	33.27	18.02	≤ **0.01**	0.004
tDCS × emotional dimension × emotional valence	5.22	0.66	1.01	0.41	0.015

tDCS = transcranial direct current stimulation ; vmPFC = ventromedial prefrontal cortex; dlPFC = left dorsolateral prefrontal cortex;; *η*_**p**_^2^ = partial eta squared; Significant results are highlighted (*p* ≤ 0.05) in bold.

**Figure 2:**
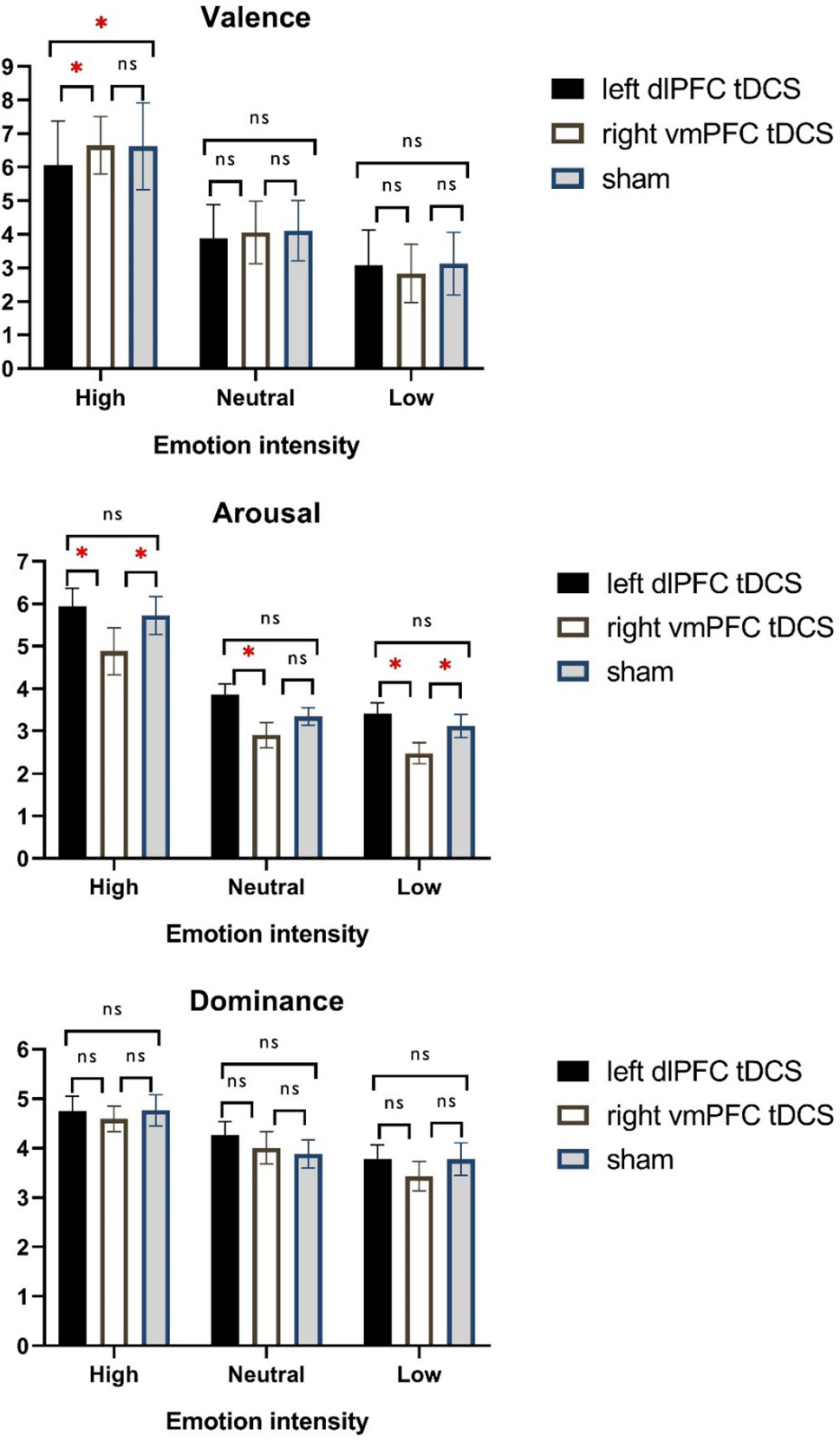
Shown are the effects of tDCS on valence, arousal and dominance of emotion ratings. *Note* vmPFC = ventromedial prefrontal cortex; dlPFC = dorsolateral prefrontal cortex; ns = non-significant; High emotional intensities represents positive, and low emotional intensities negative emotions for valence. * = indicates significant pairwise comparisons between stimulation conditions based on the results of post-hoc t-tests (paired, *p* < 0.05) n = 22; all error bars indicate Standerd Error of Mean (SEM).

More importantly, the interaction of stimulation condition × emotional dimension (*F*_3.43_ = 4.18, *p* = 0.006, partial η2 = 0.483) was significant, indicating that tDCS effects on performance were dependent on the specific emotional dimension. Follow up *post-hoc* comparisons using Sidak adjustment showed that for the dlPFC stimulation, compared to sham condition, the valence of the pictures was significantly rated less positive only for high-valenced positive pictures. When compared to the sham stimulation condition, only anodal dlPFC tDCS (*t* = 2.33, *p* = 0.036), but not anodal vmPFC tDCS (*t* = 0.12, *p* = 0.999) significantly reduced numerical ratings of only high-valenced (emotionally positive) stimuli. Furthermore, *post-hoc* comparisons using Sidak adjustment was analyzed and for tDCS over the vmPFC compared to dlPFC stimulation, a significant diminishing effect of vmPFC tDCS on all levels of arousal ratings (i.e., high, low, neutral) was found (vmPFC tDCS vs dlPFC tDCS: *t*_*high*_ = 3.15, *p* = 0.014; *t*_*medium*_ = 3.88, *p* = 0.003; *t*_*low*_ = 3.54, *p* = 0.006). In comparison with the sham condition, a significant difference between vmPFC tDCS was found only for picture with low arousal (*t* = 2.83, *p* = 0.030), but not for high arousal (*t* = 2.40, *p* = 0.075) and medium arousal (*t* = 1.87, *p* = 0.207) was observed. Anodal dlPFC tDCS however did not result in significant differences as compared with sham tDCS, in pictures with high (*t* = 1.06, *p* = 0.653), low (*t* = 0.87, *p* = 0.775) and neutral (*t* = 2.06, *p* = 0.147) arousal. With regard to emotional dominance, no significant differences were found between the active stimulation protocols (dlPFC vs vmPFC), and between the respective active, and sham protocols (Fig. 2). A significant interaction of emotional dimension × emotional valence (*F*_3.31_ = 18.02, *p* = 0.001, partial η2 = 0.702) was found as well. We found no significant interaction of tDCS × emotional dimension × emotional valence (*F*_3.46_ = 1.01, *p* = 0.414). Results are displayed in Table 3.

## Discussion

The results of this study suggest a discernable contribution of the dlPFC and vmPFC on the differential emotional evaluation of visual stimuli. Valence and arousal of emotional stimuli were modulated by application of anodal tDCS over the dlPFC and vmPFC, respectively. The dominance of emotional stimuli was not altered by any stimulation condition.

In detail, anodal tDCS over the dlPFC altered valence attribution to emotional pictures. Specifically, during anodal tDCS over the left dlPFC, the picture valence was rated lower (selectively for high-valence or positive stimuli), as compared to the sham condition only. In other words, increasing the excitability of dlPFC led to modulation of interpretation bias in valence attribution to the positive stimuli. Some studies however, with relatively similar tasks, found that anodal tDCS over the dlPFC resulted in more positive ratings of negative stimuli^50, 67^. Furthermore, this effect of tDCS has been used for amelioration of depressive symptoms^25, 68^. While the reason for these opposing effects with regard to emotional quality is unclear at present, they share a common component, which is the reduction on non-neutral emotional qualities by the dlPFC activation. Accordingly, the dlPFC reduces the valence of high- valence/ positive stimuli and increases the valence of low- valence / negative stimuli, and has thus a balancing effect. Given the negative interpretation bias in individual with depression^69^, balancing this bias resulted in a positive effect. This modulatory effect has been reported in treatment of individuals with depression through psychotherapy^70^ and cognitive rehabilitation^71, 72^.

Furthermore, a significant effect of anodal tDCS over the vmPFC was found for the arousal attribution to emotional stimuli. The arousal level attributed to emotional stimuli was reduced in the vmPFC tDCS condition. Thus the results of the present study imply that the dlPFC is closely involved in balancing the value of emotions, while the vmPFC is relevant for the arousal aspect of emotional stimuli.

It is however worth mentioning that arousal and valence are two interwoven dimensions of an emotional stimulus. Some tDCS- studies using ratings tasks found a role of the dlPFC in arousal ratings^73^, and a role of the vmPFC in valence ratings^74^. One reason for such mixed results might be methodological differences, especially with respect to task characteristics. Although these studies used ratings tasks, they did not explicitly differentiate between valence and arousal at the level of task instruction or stimuli. For instance, Freeser et al. (2014) applied an arousal ratings task after the participants underwent cognitive reappraisal strategies to down- or upregulate negative emotions. They found higher arousal ratings in the upregulation condition and lower arousal ratings in the downregulation condition after applying anodal tDCS over the right dlPFC^73^. Given the instruction of this study, up-/downregulation of an emotional stimulus is however related to the over-/ underestimation of its valence. This priming intervention, which involves manipulation of valence, likely influences the arousal ratings. Notably, the different results between that and the present study might also be caused by the fact that in the Feeser et al. study the right dlPFC was targeted for anodal tDCS, whereas in the present study the left dlPFC was the target. Furthermore, a relevance of the vmPFC has been identified for valence ratings. For instance, a tDCS study used a categorical approach of emotion ratings, and found a role of the vmPFC in happy faces, compared with fearful faces^74^. In that study, although happiness is a valence- related category of emotion, each emotional face had a mixed and uncontrolled level of arousal and valence, which makes it impossible to disentangle the relative contribution of these dimensions on the outcome. Thus these results are not in principle opposition to the findings of the present study.

For dominance as the third dimension, the different intervention conditions did not affect the ratings scores. In contrast to the well understood neural correlates of both, arousal and valence^75, 76^, the neural correlates of dominance are understudied and not well understood. This paucity of research might be caused by the relatively minor contribution of dominance to emotional ratings. While valence and arousal explain more than 50% of the variance in ratings of emotional experiences, dominance has a contribution of about 15%^62^. Accordingly, most dimensional studies are based on a two-dimensional model of emotion restricted to arousal and valence. One fMRI study describes a role of the bilateral anterior insula in dominance ratings^77^. Given the validity of these findings, a null effect of interventions of the present study on dominance ratings would make sense, because the respective tDCS protocols did not tackle the insula.

The main implication of our findings is thus a discernable contribution of dlPFC and vmPFC in specific aspects of emotional processing, implying that the dlPFC is a valence-sensitive, and the vmPFC an arousal-sensitive region. This dimension-specific contribution has been suggested by neuroimaging studies. The medial prefrontal cortex, and also the amygdala, were shown to be activated during arousal ratings in a functional magnetic resonance imaging (fMRI) study^36^. Another fMRI study has shown that regardless of valence, the amygdala, dorsomedial prefrontal cortex, and vmPFC respond equally to high-arousal pictures and words^76^. In contrast, dlPFC activity was described in high-valenced emotional experiences by an fMRI study^78^. Hence, a respective distinction of arousal- and valence- sensitive brain areas is supported by the results of these studies.

The results of this study are furthermore in accordance which concepts suggesting different processing modes related to arousal and valence^79^, which are specifically related to medial and dorsolateral regions of the prefrontal cortex. Arousal-related information is likely processed automatically and unconsciously to guide attentional resources to emotional stimuli for rapid evaluation of incoming information^13^. Several subcortical areas, including the amygdala and nucleus accumbens, are involved in arousal processing and controlled by medial prefrontal regions, including the vmPFC^80, 81^.

In contrast, valence attribution as a higher cognitive function requires conscious awareness of respective stimuli. The dlPFC, as a cognitive controller of emotion, is involved in valence-related and executive- related emotional processing, such as affective/reward processes^31, 82^, craving modulation^83^, resistance to frustration^84^, and inhibition of impulsive emotional responses^85^. In sum, the perception of arousal might be considered as an early and automatic component of emotional processing, mostly related to vmPFC activation, whereas perception of valence attribution as a late and cognitive component of emotional processing is mediated by the dlPFC.

A respective discernable contribution of dlPFC and vmPFC in emotional processing is also supported by the psychopathology of clinical syndromes, such as anxiety and depression. According to the two stage theory of depression and anxiety developed by Williams^86^, anxiety is related to early information processing and attention bias to threat-related stimuli. In contrast, depression is related to abnormal late information processing such as elaboration and negative interpretation of information. From a neurophysiological perspective, hypoactivity of the dlPFC in depression^87^ and hypoactivity of the vmPFC in anxiety^88^ confirms this concept. Recent studies in healthy humans also show that increasing vmPFC activity enhances fear extinction which has implications for anxiety disorders^89, 90^. Correspondingly, anxious individuals are affected by an abnormal function of the arousal dimension of emotional experience^91^, while in depressed individuals the valence dimension of emotional experience is negatively biased^92^.

With respect to psychotherapeutic approaches, correction of valence overestimation is a therapeutic goal in depression. For example, cognitive behavioral therapy downregulates neural activity during emotion regulation and modulates valence overestimation^93^. In contrast, the modulation of arousal is a primary therapeutic goal in the treatment of anxiety syndromes, namely exposition training^94^.

The results of our study do not only confirm a relevant role of both prefrontal areas in emotion processing, they furthermore support a specific function of these areas in eliminating, controlling, or diminishing arousal and valence associated with emotional experiences, which is critical for normal reactions to emotional experiences. Indeed, impairment or dysregulation of this control leads to psychopathologic states^95^. The respective emotion control mechanisms might however differ between vmPFC and dlPFC. The vmPFC, in a basic manner, is involved in extinction for emotional control^96, 97^, while the dlPFC is relevant for cognitively more complex regulation strategies^98^.

In this line, increased activation of the vmPFC during extinction has been shown by neuroimaging studies^99^, and cortical thickness of vmPFC predicts the rate of extinction^97^. Moreover, extinction deficits have been reported in anxiety^95^, and PTSD^100^. In further accordance, exposition therapies used for phobias, and anxiety disorders focus mainly on alteration of the arousal dimension of emotional stimuli, and increase of vmPFC excitability through repetitive transcranial magnetic stimulation leads to symptom improvement in individuals with anxiety^101^.

**Figure 3:**
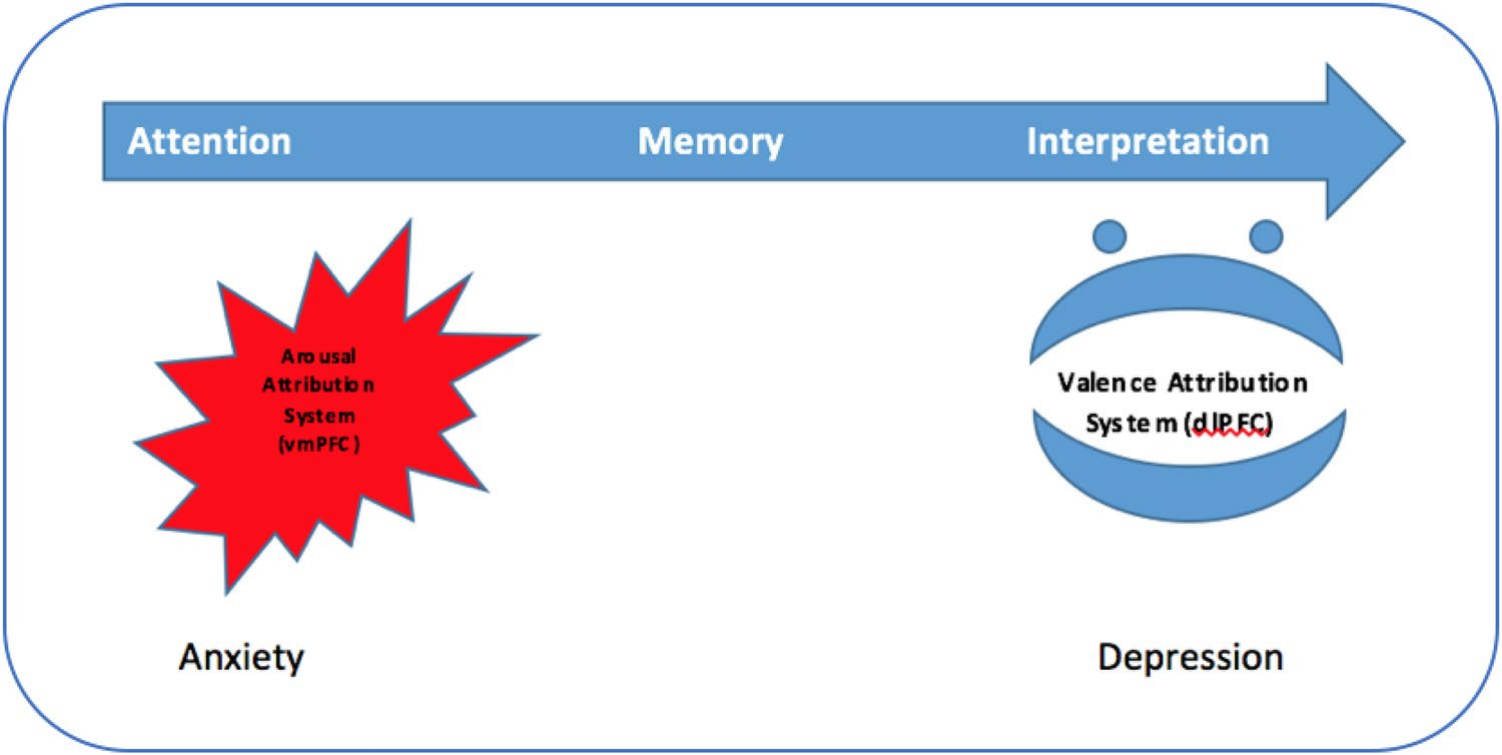
Emotional attribution system disorders in depression and anxiety. A schematic diagram for the assumed role of arousal and valence in the psychopathology of anxiety and depression. Abbreviations: dlPFC: dorsolateral prefrontal cortex, vmPFC: ventromedial prefrontal cortex.

On the other hand, the dlPFC is crucial for valence attribution to emotional experience. This includes cognitive regulation strategies^102^, such as suppression, attention redirection, or reappraisal strategies^103^. For example, reappraisal is used to reinterpret a picture in a less or more negative context^102^. Impaired emotional regulation due to the dlPFC dysfunction is closely associated with depression, in which the interaction of cognition and emotion is largely disturbed. Furthermore, executive dysfunctions, which involve crucially the dlPFC, play an important role in depression^104, 105^. This also explains why excitatory brain stimulation over the dlPFC is well-suited to reduce depressive symptoms and states^25, 106, 107^ (Fig. 3).

At last, the results of the present study suggest that an association of emotions with specific prefrontal brain areas can be appropriately explored with the dimensional approach. In another approach to categorize emotions, the categorical approach, 6 basic emotions are considered as main emotional states, including sadness, happiness, anger, fear, disgust, and surprise^108^. Some studies consider this categorical approach for the study of neural correlates of emotions. For instance, a role of the vmPFC in perception of fear, disgust, and anger^109^, but also in the perception of positive emotions^110^ has been identified. Based on the findings of the present study, respective findings are not contradictory, if one assumes that the common factor, which is relevant for positive and negative emotions, and for which vmPFC is relevant, is the dimension of arousal.

Some limitations of this study should be taken into account. In the present study, as usual in studies of emotional dimensions, we asked the participants to rate the pictures based on a self-assessment manikin, explicitly. At least for arousal, which is to a relevant degree processed implicitly, it would have been advantageous to add electrophysiological recordings, which take this into account. We thus propose that future studies should add recording of somatic markers such as heart rate, galvanic skin response or pupil diameter for evaluation of arousal.

Each picture was presented three times for ratings of valence, arousal, and dominance in identical order throughout the experiment. Therefore, we cannot rule out that a familiarity effect was present for the later ratings, due to the identical order of the respective ratings. Because the ratings at different presentation time points were however uniformly dedicated to the respective dimensions, and identical between sessions, it is unlikely that such a possible order effect would have had a discernable effect on the respective ratings between intervention sessions. In future studies, it would however be preferable to use a counterbalanced order of dimension ratings to enable the analysis of the effect of order of presentation.

Methodologically, our study had an exploratory design. The results should thus be confirmed in larger sample sizes. Finally, a one-to-one transferability of the study results obtained in healthy participants to clinical populations of interest, including anxiety disorders, phobias, and depression, cannot be taken for granted. It would, however, be important to explore respective mechanisms also in these patient populations, because it might help to identify promising targets for future intervention approaches, including brain stimulation.

## References

[1] Kolb B, Whishaw IQ (2009). Fundamentals of Human Neuropsychology.

[2] Kane MJ, Engle RW (2002). The role of prefrontal cortex in working-memory capacity, executive attention, and general fluid intelligence: an individual-differences perspective. Psychon. Bull. Rev..

[3] Stuss DT, Benson DF (1986). The Frontal Lobes.

[4] Ghanavati E, Salehinejad MA, Nejati V, Nitsche MA (2019). Differential role of prefrontal, temporal and parietal cortices in verbal and figural fluency: Implications for the supramodal contribution of executive functions. Sci. Rep..

[5] Barbey AK, Koenigs M, Grafman J (2013). Dorsolateral prefrontal contributions to human working memory. Cortex.

[6] Liu S (2015). Brain activity and connectivity during poetry composition: toward a multidimensional model of the creative process. Hum. Brain Mapp..

[7] Vossel S, Geng JJ, Fink GR (2014). Dorsal and ventral attention systems: distinct neural circuits but collaborative roles. The Neuroscientist.

[8] Rahnev D, Nee DE, Riddle J, Larson AS, D’Esposito M (2016). Causal evidence for frontal cortex organization for perceptual decision making. Proc. Natl. Acad. Sci..

[9] Proudfit GH (2015). The reward positivity: from basic research on reward to a biomarker for depression. Psychophysiology.

[10] Kim H, Shimojo S, O’doherty JP (2010). Overlapping responses for the expectation of juice and money rewards in human ventromedial prefrontal cortex. Cereb. Cortex.

[11] Camille N (2004). The involvement of the orbitofrontal cortex in the experience of regret. Science.

[12] Pujara MS, Philippi CL, Motzkin JC, Baskaya MK, Koenigs M (2016). Ventromedial prefrontal cortex damage is associated with decreased ventral striatum volume and response to reward. J. Neurosci..

[13] Salehinejad MA, Nejati V, Nitsche MA (2019). Neurocognitive correlates of self-esteem: from self-related attentional bias to involvement of the ventromedial prefrontal cortex. Neurosci. Res..

[14] Lyons, W. The philosophy of cognition and emotion. *Handbook of cognition and emotion*, 21–44 (1999).

[15] Zajonc RB (1980). Feeling and thinking: preferences need no inferences. Am. Psychol..

[16] Lazarus, R. S. On the primacy of cognition. (1984).

[17] Steele JD, Lawrie S (2004). Segregation of cognitive and emotional function in the prefrontal cortex: a stereotactic meta-analysis. Neuroimage.

[18] Öhman A, Flykt A, Esteves F (2001). Emotion drives attention: detecting the snake in the grass. J. Exp. Psychol. Gen..

[19] Kim SH, Hamann S (2007). Neural correlates of positive and negative emotion regulation. J. Cogn. Neurosci..

[20] Ray RD, Wilhelm FH, Gross JJ (2008). All in the mind's eye? Anger rumination and reappraisal. J. Pers. Soc. Psychol..

[21] Borhani K, Nejati V (2018). Emotional face recognition in individuals withattention-deficit/hyperactivity disorder: a review article. Dev. Neuropsychol..

[22] O’Reilly RC (2010). The what and how of prefrontal cortical organization. Trends Neurosci..

[23] Sallet J (2013). The organization of dorsal frontal cortex in humans and macaques. J. Neurosci..

[24] Alizadehgoradel J (2020). Repeated stimulation of the dorsolateral-prefrontal cortex improves executive dysfunctions and craving in drug addiction: a randomized, double-blind, parallel-group study. Brain Stimul..

[25] Salehinejad MA, Ghanavai E, Rostami R, Nejati V (2017). Cognitive control dysfunction in emotion dysregulation and psychopathology of major depression (MD): evidence from transcranial brain stimulation of the dorsolateral prefrontal cortex (DLPFC). J. Affect. Disord..

[26] Hakamata Y (2010). Attention bias modification treatment: a meta-analysis toward the establishment of novel treatment for anxiety. Biol. Psychiat..

[27] Hartley CA, Phelps EA (2010). Changing fear: the neurocircuitry of emotion regulation. Neuropsychopharmacology.

[28] Nejati V, Salehinejad MA, Sabayee A (2018). Impaired working memory updating affects memory for emotional and non-emotional materials the same way: evidence from post-traumatic stress disorder (PTSD). Cogn. Process..

[29] Watkins E, Brown R (2002). Rumination and executive function in depression: An experimental study. J. Neurol. Neurosurg. Psychiatry.

[30] Koenigs M, Grafman J (2009). The functional neuroanatomy of depression: distinct roles for ventromedial and dorsolateral prefrontal cortex. Behav. Brain Res..

[31] Nejati V, Salehinejad MA, Nitsche MA (2018). Interaction of the left dorsolateral prefrontal cortex (l-DLPFC) and right orbitofrontal cortex (OFC) in hot and cold executive functions: evidence from transcranial direct current stimulation (tDCS). Neuroscience.

[32] Gray JR (2001). Emotional modulation of cognitive control: approach–withdrawal states double-dissociate spatial from verbal two-back task performance. J. Exp. Psychol. Gen..

[33] Van Reekum CM (2007). Individual differences in amygdala and ventromedial prefrontal cortex activity are associated with evaluation speed and psychological well-being. J. Cogn. Neurosci..

[34] Heller AS (2013). Sustained striatal activity predicts eudaimonic well-being and cortisol output. Psychol. Sci..

[35] Perlstein WM, Elbert T, Stenger VA (2002). Dissociation in human prefrontal cortex of affective influences on working memory-related activity. Proc. Natl. Acad. Sci..

[36] Phan KL (2003). Activation of the medial prefrontal cortex and extended amygdala by individual ratings of emotional arousal: a fMRI study. Biol. Psychiat..

[37] Polanía R, Nitsche MA, Ruff CC (2018). Studying and modifying brain function with non-invasive brain stimulation. Nat. Neurosci..

[38] Vicario CM, Salehinejad MA, Felmingham K, Martino G, Nitsche MA (2019). A systematic review on the therapeutic effectiveness of non-invasive brain stimulation for the treatment of anxiety disorders. Neurosci. Biobehav. Rev..

[39] Nitsche M (2012). Effects of frontal transcranial direct current stimulation on emotional state and processing in healthy humans. Front. Psychiatry.

[40] Salehinejad MA, Wischnewski M, Nejati V, Vicario CM, Nitsche MA (2019). Transcranial direct current stimulation in attention-deficit hyperactivity disorder: a meta-analysis of neuropsychological deficits. PLoS ONE.

[41] Ghanavati E, Nejati V, Salehinejad MA (2018). Transcranial direct current stimulation over the posterior parietal cortex (PPC) enhances figural fluency: implications for creative cognition. J. Cogn. Enhanc..

[42] Kuo, M.-F., Polanía, R. & Nitsche, M. in *Transcranial Direct Current Stimulation in Neuropsychiatric Disorders: Clinical Principles and Management* (eds André Brunoni, Michael Nitsche, & Colleen Loo) 29–46 (Springer International Publishing, 2016).

[43] Salehinejad MA, Ghanavati E (2020). Complexity of cathodal tDCS: relevance of stimulation repetition, interval, and intensity. J. Physiol..

[44] Nitsche MA, Paulus W (2000). Excitability changes induced in the human motor cortex by weak transcranial direct current stimulation. J. Physiol..

[45] Nitsche MA, Paulus W (2001). Sustained excitability elevations induced by transcranial DC motor cortex stimulation in humans. Neurology.

[46] Vanderhasselt M-A (2013). tDCS over the left prefrontal cortex enhances cognitive control for positive affective stimuli. PLoS ONE.

[47] Nitsche MA (2012). Effects of frontal transcranial direct current stimulation on emotional state and processing in healthy humans. Front. Psychiatry.

[48] Cattaneo Z (2014). The world can look better: enhancing beauty experience with brain stimulation. Soc. Cogn. Affect. Neurosci..

[49] Maeoka H, Matsuo A, Hiyamizu M, Morioka S, Ando H (2012). Influence of transcranial direct current stimulation of the dorsolateral prefrontal cortex on pain related emotions: a study using electroencephalographic power spectrum analysis. Neurosci. Lett..

[50] Pena-Gomez C, Vidal-Pineiro D, Clemente IC, Pascual-Leone A, Bartres-Faz D (2011). Down-regulation of negative emotional processing by transcranial direct current stimulation: effects of personality characteristics. PLoS ONE.

[51] Sanchez-Lopez A, Vanderhasselt M-A, Allaert J, Baeken C, De Raedt R (2018). Neurocognitive mechanisms behind emotional attention: Inverse effects of anodal tDCS over the left and right DLPFC on gaze disengagement from emotional faces. Cogn. Affect. Behav. Neurosci..

[52] Grimm S (2008). Imbalance between left and right dorsolateral prefrontal cortex in major depression is linked to negative emotional judgment: an fMRI study in severe major depressive disorder. Biol. Psychiatry.

[53] Allaert J, Sanchez-Lopez A, De Raedt R, Baeken C, Vanderhasselt M-A (2019). Inverse effects of tDCS over the left versus right DLPC on emotional processing: a pupillometry study. PLoS ONE.

[54] Price AR, Hamilton RH (2015). A re-evaluation of the cognitive effects from single-session transcranial direct current stimulation. Brain Stimul..

[55] Imburgio MJ, Orr JM (2018). Effects of prefrontal tDCS on executive function: methodological considerations revealed by meta-analysis. Neuropsychologia.

[56] Salehinejad MA, Ghayerin E, Nejati V, Yavari F, Nitsche MA (2020). Domain-specific involvement of the posterior parietal cortex in attention network and attentional control of ADHD: a randomized, cross-over, sham-controlled tDCS study. Neuroscience.

[57] Salehinejad MA (2020). Transcranial direct current stimulation in ADHD: a systematic review of efficacy, safety, and protocol-induced electrical field modeling results. Neurosci. Bull..

[58] Moos K, Vossel S, Weidner R, Sparing R, Fink GR (2012). Modulation of Top-down control of visual attention by cathodal tDCS over right IPS. J. Neurosci..

[59] Kajimura S, Kochiyama T, Nakai R, Abe N, Nomura M (2016). Causal relationship between effective connectivity within the default mode network and mind-wandering regulation and facilitation. NeuroImage.

[60] Faul, F., Erdfelder, E., Buchner, A. & Lang, A. (2013).

[61] Minarik T (2016). The importance of sample size for reproducibility of tDCS effects. Front. Hum. Neurosci..

[62] Bradley MM, Lang PJ (1994). Measuring emotion: the self-assessment manikin and the semantic differential. J. Behav. Ther. Exp. Psychiatry.

[63] Lang, P. J., Bradley, M. M. & Cuthbert, B. N. International affective picture system (IAPS): Technical manual and affective ratings. *NIMH Center for the Study of Emotion and Attention*, 39–58 (1997).

[64] Stagg CJ (2013). Widespread modulation of cerebral perfusion induced during and after transcranial direct current stimulation applied to the left dorsolateral prefrontal cortex. J. Neurosci..

[65] Zheng H (2016). Modulating the activity of ventromedial prefrontal cortex by anodal tDCS enhances the trustee’s repayment through altruism. Front. Psychol..

[66] Palm U (2013). Evaluation of sham transcranial direct current stimulation for randomized, placebo-controlled clinical trials. Brain Stimul..

[67] Boggio PS, Zaghi S, Fregni F (2009). Modulation of emotions associated with images of human pain using anodal transcranial direct current stimulation (tDCS). Neuropsychologia.

[68] Molavi, P. *et al.* Repeated transcranial direct current stimulation of dorsolateral-prefrontal cortex improves executive functions, cognitive reappraisal emotion regulation, and control over emotional processing in borderline personality disorder: a randomized, sham-controlled, parallel-group study. *J. Affect. Disord.* (2020). 10.1016/j.jad.2020.05.007 32469838

[69] Nejati V (2018). Negative interpretation of social cue in depression: Evidence from reading mind from eyes test. Neurol. Psychiatry Brain Res..

[70] Ajilchi B, Kisely S, Nejati V, Frederickson J (2020). Effects of intensive short-term dynamic psychotherapy on social cognition in major depression. J. Mental Health.

[71] Roshani F, Nejati V, Fathabadi J (2020). Effect of interpretation bias modification on remediation of behavioral and cognitive symptoms in depression. J. Psychol. Sci..

[72] Nejati V, Fathi E, Shahidi S, Salehinejad MA (2019). Cognitive training for modifying interpretation and attention bias in depression: relevance to mood improvement and implications for cognitive intervention in depression. Asian J. Psychiatry.

[73] Feeser M, Prehn K, Kazzer P, Mungee A, Bajbouj M (2014). Transcranial direct current stimulation enhances cognitive control during emotion regulation. Brain stimulation.

[74] Winker C (2018). Noninvasive stimulation of the ventromedial prefrontal cortex modulates emotional face processing. Neuroimage.

[75] Anders S, Lotze M, Erb M, Grodd W, Birbaumer N (2004). Brain activity underlying emotional valence and arousal: a response-related fMRI study. Hum. Brain Mapp..

[76] Kensinger EA, Schacter DL (2006). Processing emotional pictures and words: effects of valence and arousal. Cogn., Affect. Behav. Neurosci..

[77] Jerram M, Lee A, Negreira A, Gansler D (2014). The neural correlates of the dominance dimension of emotion. Psychiatry Res. Neuroimaging.

[78] Colibazzi T (2010). Neural systems subserving valence and arousal during the experience of induced emotions. Emotion.

[79] Posner J, Russell JA, Peterson BS (2005). The circumplex model of affect: an integrative approach to affective neuroscience, cognitive development, and psychopathology. Dev. Psychopathol..

[80] Kringelbach ML, O’Doherty J, Rolls ET, Andrews C (2003). Activation of the human orbitofrontal cortex to a liquid food stimulus is correlated with its subjective pleasantness. Cereb. Cortex.

[81] Soder HE, Potts GF (2018). Medial frontal cortex response to unexpected motivationally salient outcomes. Int. J. Psychophysiol..

[82] Strobach T (2018). Modulation of dual-task control with right prefrontal transcranial direct current stimulation (tDCS). Exp. Brain Res..

[83] Shahbabaie A (2014). State dependent effect of transcranial direct current stimulation (tDCS) on methamphetamine craving. Int. J. Neuropsychopharmacol..

[84] Salehinejad MA, Nejati V, Derakhshan M (2017). Neural correlates of trait resiliency: evidence from electrical stimulation of the dorsolateral prefrontal cortex (dLPFC) and orbitofrontal cortex (OFC). Pers. Individ. Differ..

[85] Coplan JD, Webler R, Gopinath S, Abdallah CG, Mathew SJ (2018). Neurobiology of the dorsolateral prefrontal cortex in GAD: aberrant neurometabolic correlation to hippocampus and relationship to anxiety sensitivity and IQ. J. Affect. Disord..

[86] Williams JMG, Watts FN, MacLeod C, Mathews A (1988). Cognitive Psychology and Emotional Disorders.

[87] Fitzgerald PB (2006). An analysis of functional neuroimaging studies of dorsolateral prefrontal cortical activity in depression. Psychiatry Res. Neuroimaging.

[88] Hiser, J. & Koenigs, M. The multifaceted role of ventromedial prefrontal cortex in emotion, decision-making, social cognition, and psychopathology. *Biol. Psychiatry* (2017). 10.1016/j.biopsych.2017.10.030 PMC5862740 29275839

[89] Vicario CM (2019). Anodal transcranial direct current stimulation over the ventromedial prefrontal cortex enhances fear extinction in healthy humans: a single blind sham-controlled study. Brain Stimul..

[90] Vicario, C. M. *et al.* Anodal transcranial direct current stimulation over the ventromedial prefrontal cortex enhances fear extinction in healthy humans: a single blind sham-controlled study. *Brain Stimul. Basic Transl. Clin. Res. Neuromodul.* (2019). 10.1016/j.brs.2019.12.022 31899172

[91] Arent SM, Landers DM (2003). Arousal, anxiety, and performance: a reexamination of the inverted-U hypothesis. Res. Q. Exerc. Sport.

[92] Groenewold NA, Opmeer EM, de Jonge P, Aleman A, Costafreda SG (2013). Emotional valence modulates brain functional abnormalities in depression: evidence from a meta-analysis of fMRI studies. Neurosci. Biobehav. Rev..

[93] Rubin-Falcone H (2018). Longitudinal effects of cognitive behavioral therapy for depression on the neural correlates of emotion regulation. Psychiatry Res. Neuroimaging.

[94] Hofmann SG, Heering S, Sawyer AT, Asnaani A (2009). How to handle anxiety: the effects of reappraisal, acceptance, and suppression strategies on anxious arousal. Behav. Res. Ther..

[95] Bremner JD (2004). Brain imaging in anxiety disorders. Expert Rev. Neurother..

[96] Phelps EA, LeDoux JE (2005). Contributions of the amygdala to emotion processing: from animal models to human behavior. Neuron.

[97] Milad MR (2005). Thickness of ventromedial prefrontal cortex in humans is correlated with extinction memory. Proc. Natl. Acad. Sci..

[98] Ochsner KN, Gross JJ (2005). The cognitive control of emotion. Trends Cogn. Sci..

[99] Milad MR (2007). Recall of fear extinction in humans activates the ventromedial prefrontal cortex and hippocampus in concert. Biol. Psychiatry.

[100] Rothbaum BO, Davis M (2003). Applying learning principles to the treatment of post-trauma reactions. Ann. N. Y. Acad. Sci..

[101] Paes F (2013). Repetitive transcranial magnetic stimulation (rTMS) to treat social anxiety disorder: case reports and a review of the literature. Clin. Pract. Epidemiol. Mental Health CP & EMH.

[102] Ochsner KN, Bunge SA, Gross JJ, Gabrieli JD (2002). Rethinking feelings: an FMRI study of the cognitive regulation of emotion. J. Cogn. Neurosci..

[103] Notzon S, Steinberg C, Zwanzger P, Junghöfer M (2018). Modulating emotion perception: Opposing effects of inhibitory and excitatory prefrontal cortex stimulation. Biol. Psychiatry Cogn. Neurosci. Neuroimaging.

[104] Ajilchi B, Nejati V (2017). Executive functions in students with depression, anxiety, and stress symptoms. Basic Clin. Neurosci..

[105] Ajilchi B, Nejati V, Town JM, Wilson R, Abbass A (2016). Effects of intensive short-term dynamic psychotherapy on depressive symptoms and executive functioning in major depression. J. Nerv. Mental Dis..

[106] Nejati V, Salehinejad MA, Shahidi N, Abedin A (2017). Corrigendum to Psychological intervention combined with direct electrical brain stimulation (PIN-CODES) for treating major depression: a pre-test, post-test, follow-up pilot study [Neurol. Psychiatry Brain Res. 25 (2017) 15–23]. Neurol. Psychiatry Brain Res..

[107] Vanderhasselt M-a, De Raedt R, Baeken C, Leyman L, D’haenen H (2009). A single session of rTMS over the left dorsolateral prefrontal cortex influences attentional control in depressed patients. World J. Biol. Psychiatry.

[108] Ekman P (1987). Universals and cultural differences in the judgments of facial expressions of emotion. J. Pers. Soc. Psychol..

[109] Lindquist KA, Wager TD, Kober H, Bliss-Moreau E, Barrett LF (2012). The brain basis of emotion: a meta-analytic review. Behav. Brain Sci..

[110] Kirkland T, Cunningham WA (2011). Neural basis of affect and emotion. Wiley Interdiscip. Rev. Cogn. Sci..

